# Structural Characterization of *Mycobacterium abscessus* Phosphopantetheine Adenylyl Transferase Ligand Interactions: Implications for Fragment-Based Drug Design

**DOI:** 10.3389/fmolb.2022.880432

**Published:** 2022-05-30

**Authors:** Sherine E. Thomas, William J. McCarthy, Jamal El Bakali, Karen P. Brown, So Yeon Kim, Michal Blaszczyk, Vítor Mendes, Chris Abell, R. Andres Floto, Anthony G. Coyne, Tom L. Blundell

**Affiliations:** ^1^ Department of Biochemistry, University of Cambridge, Cambridge, United Kingdom; ^2^ Yusuf Hamied Department of Chemistry, University of Cambridge, Cambridge, United Kingdom; ^3^ MRC Laboratory of Molecular Biology, Molecular Immunity Unit, Department of Medicine, University of Cambridge, Cambridge, United Kingdom; ^4^ Cambridge Centre for Lung Infection, Royal Papworth Hospital, Cambridge, United Kingdom

**Keywords:** *Mycobacterium abscessus*, *Mycobacterium tuberculosis*, Coenzyme A pathway, CoaD/ PPAT, drug discovery, fragment-based, antibiotics

## Abstract

Anti-microbial resistance is a rising global healthcare concern that needs urgent attention as growing number of infections become difficult to treat with the currently available antibiotics. This is particularly true for mycobacterial infections like tuberculosis and leprosy and those with emerging opportunistic pathogens such as *Mycobacterium abscessus*, where multi-drug resistance leads to increased healthcare cost and mortality. *M. abscessus* is a highly drug-resistant non-tuberculous *mycobacterium* which causes life-threatening infections in people with chronic lung conditions such as cystic fibrosis. In this study, we explore *M. abscessus* phosphopantetheine adenylyl transferase (PPAT), an enzyme involved in the biosynthesis of Coenzyme A, as a target for the development of new antibiotics. We provide structural insights into substrate and feedback inhibitor binding modes of *M. abscessus* PPAT, thereby setting the basis for further chemical exploration of the enzyme. We then utilize a multi-dimensional fragment screening approach involving biophysical and structural analysis, followed by evaluation of compounds from a previous fragment-based drug discovery campaign against *M. tuberculosis* PPAT ortholog. This allowed the identification of an early-stage lead molecule exhibiting low micro molar affinity against *M. abscessus* PPAT (K_d_ 3.2 ± 0.8 µM) and potential new ways to design inhibitors against this enzyme. The resulting crystal structures reveal striking conformational changes and closure of solvent channel of *M. abscessus* PPAT hexamer providing novel strategies of inhibition. The study thus validates the ligandability of *M. abscessus* PPAT as an antibiotic target and identifies crucial starting points for structure-guided drug discovery against this bacterium.

## Introduction

The emergence of highly drug-resistant non-tuberculous mycobacteria (NTM) in recent years has intensified the challenges to clinically manage chronic lung conditions such as cystic fibrosis (Nessar et al., 2012; Bryant et al., 2021). *Mycobacterium abscessus* is one of the most common NTMs found in cystic fibrosis (CF) related lung infections and being intrinsically resistant to most antibiotics, causes very high rates of lung function decline (Nessar et al., 2012; Stephenson et al., 2017). Although previously thought to be a benign environmental microbe, *M. abscessus* is increasingly observed as a cause of chronic lung and soft-tissues infections, usually in the context of lung diseases like CF and in immunocompromised individuals (Bar-On et al., 2015; Parkins and Floto, 2015). *M. abscessus* is highly challenging to treat due to uniform resistance to standard anti-tuberculosis drugs, in addition to most antibiotics. High virulence and resistance to chemotherapy of this bacterium is attributed to a combination of intrinsic and acquired resistance mechanisms (Hill et al., 2012). Current clinical management of *M. abscessus* involves prolonged therapy using antibiotic combinations such as amikacin, cefoxitin and macrolides which are poorly tolerated, often resulting in therapeutic failure (Yang et al., 2017). This NTM is also associated with life-threatening disseminated infection in lung transplant recipients (Hill et al., 2012). Thus, there is an urgent need for effective and less toxic drugs to treat *M. abscessus* infections.

**FIGURE 1:**
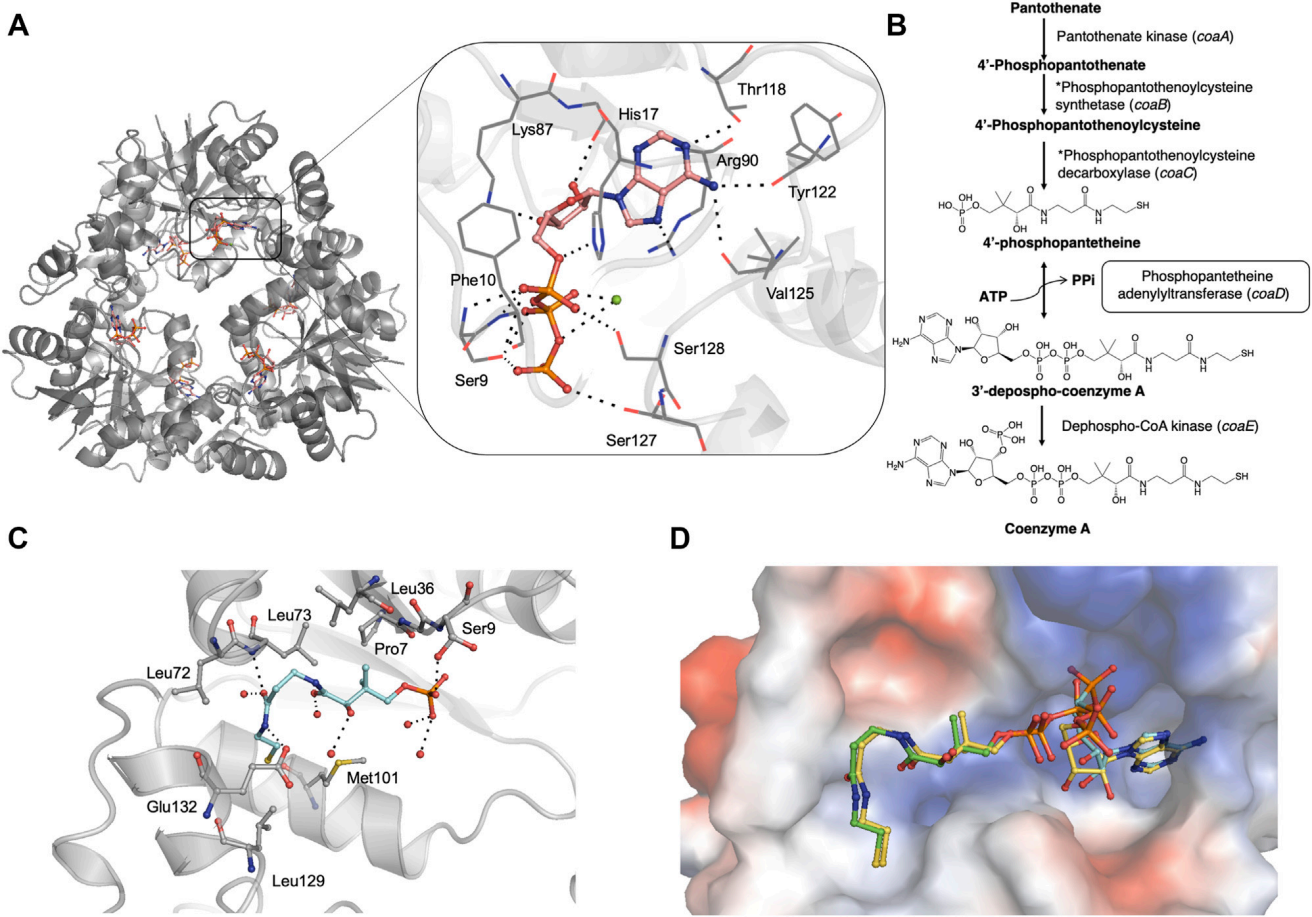
**(A)** Crystal structure of *M. abscessus* PPAT hexamer (grey) in complex with ATP (PDB code 7YWM). The detailed interaction map is also shown with ATP in salmon stick model and interacting residues in grey line representation. Polar hydrogen bond contacts are shown in black dotted lines and active site Mg^2+^ as green sphere. **(B)** Biosynthetic pathway of coenzyme A in bacteria. The step catalyzed by PPAT enzyme is highlighted in black box. Corresponding *E. coli* gene names are given in brackets. *These two steps are catalyzed by a single polypeptide in bacteria. **(C)** *Mab* PPAT in complex with 4′-phosphopantetheine (PhP) (PDB code 7YY0) showing interacting residues in grey and PhP in blue stick model. **(D)** Crystal structure of *M. abscessus* PPAT in ternary complex with substrates, 4′-phosphopantetheine (PhP) and non-hydrolysable ATP analogue AMPCPP, solved at 1.62 Å (PDB code 7YY1). PhP is shown in green, AMPCPP in blue stick models. *Mab* PPAT active site is shown as a space-filling model coloured according to surface electrostatics. Structural superposition with *Mab* PPAT:dpCoA (yellow stick model) shows close agreement in the positions of substrates and product.

Coenzyme A (CoA) is an essential cellular co-factor that acts as an important acyl-group carrier in all organisms. CoA plays a key role in mediating numerous biosynthetic, degradative, and metabolic pathways (Geerlof et al., 1999). The biosynthesis of CoA consists of a five-step reaction involving pantothenate (Vitamin B5), cysteine and ATP as starting substrates (Abiko, 1967). Phosphopantetheine adenylyltransferase (PPAT or CoaD) catalyses the penultimate step in the biosynthesis of CoA in prokaryotes. The enzyme catalyses the reversible transfer of an adenylyl group from ATP to 4′-phosphopantetheine (PhP) to yield 3′-dephospho-CoA (dpCoA) and pyrophosphate. The product dpCoA in turn is phosphorylated by dephospho-CoA kinase (DPCK/CoaE) to generate the final product of the pathway, Coenzyme A (Robishaw and Neely, 1985; Izard and Geerlof, 1999) (Figure 1B).

Enzymes in the CoA biosynthetic pathway have been recognised as attractive antibiotic targets (Cole et al., 1998). Previous studies on CoA pathway intermediates in *E. coli* show that pantothenate and 4′-phosphopantetheine (PhP) accumulate in the cell, suggesting an important rate-limiting role of PPAT in the pathway for regulating the cellular content of CoA (Jackowski and Rock, 1984; Gerdes et al., 2002; Wubben and Mesecar, 2011). Further studies on *M. tuberculosis* show the essential role played by the *coaD* gene in mycobacterial growth *in vitro*, confirming the potential of PPAT as an antibiotic target (Ambady et al., 2012) (El Bakali et al., 2020). In higher eukaryotes, the final two steps of CoA biosynthesis are catalysed by a single bi-functional enzyme, CoA synthase, containing a PPAT like domain. The marked structural differences between bacterial and human PPAT domain architectures further facilitate target specific antibiotic discovery without inducing mechanism-based toxicity (Aghajanian and Worrall, 2002; Daugherty et al., 2002; Zhyvoloup et al., 2002).

Previous attempts to develop inhibitors targeting *E. coli* PPAT enzyme, using combinatorial synthesis approaches, were only partially successful as the resulting candidate compounds showed poor inhibitory activity in whole cell-based assays (Zhao et al., 2003). A high throughput screening (HTS) campaign against *E. coli* PPAT screened 750,000 compounds, resulting in the identification of 99 validated hits. Some of these hits were developed into compounds exhibiting low micro molar IC_50_ against *E. coli* PPAT (Miller et al., 2010), however further structural biology evidence is needed to establish the ligand binding mode. Yet another HTS of the AstraZeneca compound library and subsequent structure-guided lead optimization, targeting PPAT from Gram-positive bacteria such as *S. pneumoniae* and *S. aureus,* led to the development of compounds that significantly reduced the bacterial burden both *in vitro* and in animal models of infection (De Jonge et al., 2013). However, these compounds were not progressed further into clinical development due to unfavourable toxicity and pharmacokinetic profiles. A recent fragment-based drug discovery campaign (Skepper et al., 2018) resulted in the development of several unique chemical scaffolds targeting the *E. coli* PPAT active site. Although some of these compounds exhibited nanomolar affinity *in vitro*, sufficient cellular potency could not be achieved against *E. coli* and other Gram-negative bacteria to warrant further chemical development of the series.

In this study, we report some of the first structural insights into the substrate and feedback inhibitor binding modes of PPAT from *M. abscessus*. We then explore the chemical space of *M. abscessus* PPAT using a multi-dimensional fragment screening approach involving biophysical and X-ray crystallographic analysis. The study is further extended by incorporating the knowledge gained from a previous fragment-based drug discovery campaign in our research groups, against *M. tuberculosis* PPAT ortholog (El Bakali et al., 2020), to identify potential early-stage lead compound targeting *M. abscessus* PPAT. Results from the study validate the ligandability of *M. abscessus* PPAT as an antibiotic target and facilitate the initiation of structure-guided drug discovery targeting this highly drug-resistant *mycobacterium*.

## Materials and Methods

### PCR and Molecular Cloning

The *coaD* gene (MAB_3259c) was amplified from *Mycobacterium abscessus* (ATCC 19977) genomic DNA using the following primers (Sigma):

Forward Primer: 5′-ATA​GGA​TCC​ATG​ACG​GGA​GCG​GTG​TGC​CC-3′ and
Reverse Primer: 5′-ACC​AAG​CTT​CTA​TTG​TGC​CTG​GCC​ACG​CAG​TTT​C -3′

The purified PCR products and pET28a SUMO vector were subjected to restriction digestion with BamHI and HindIII restriction endonucleases (ThermoScientific). The ligation of digested insert and vector was done using T4 DNA ligase (New England Biolabs) by incubation at room temperature for 10 min. The ligation product was transformed into *E. coli* DH5α competent cells by the heat-shock method, plated on LB agar-kanamycin plates and incubated at 37°C. Single colonies were randomly picked on the following day and inoculated in LB media with kanamycin (30 μg/ml) and grown overnight at 37°C. Plasmids from the resulting cultures were isolated, purified and the integrity of the clones was confirmed by sequencing (DNA Sequencing Facility, Department of Biochemistry, Cambridge).

### Expression and Purification of Full-Length *Mab* PPAT

*E. coli* BL21 (DE3) strain containing pET28a N-His SUMO-PPAT plasmid was grown overnight at 37°C in LB-media containing Kanamycin (30 μg/ml). This seed stage culture was used to inoculate 6 L of 2x YT media with Kanamycin (30 μg/ml) until optical density (A_600nm_) reached 0.6. The expression of recombinant construct was induced by the addition of Isopropyl β-D-1-thiogalactopyranoside (IPTG) to a final concentration of 0.5 mM and further allowed to grow at 18°C for 18 h. Cells were harvested by centrifugation at 4°C for 20 min at 5,000 g and the pellet was re-suspended in buffer A (50 mM Tris-HCl pH 8.0, 350 mM NaCl, 20 mM Imidazole). 10 μg/ml DNaseI, 5 mM MgCl_2_ and 3 protease inhibitor cocktail tablets (New England Biolabs) were added to the cell suspension. The cells were lysed by sonication (Branson). The lysate clarified by centrifugation at 4°C for 40 min at 25,568 g, was passed through a pre-equilibriated (with buffer A), 10 ml pre-packed Nickel-sepharose column (HiTrap IMAC FF, GE Healthcare). The column was washed with buffer A and the bound protein was eluted using buffer B (50 mM Tris-HCl pH 8.0, 350 mM NaCl and 500 mM Imidazole). Eluates from HiTrap IMAC column were pooled and Ulp1 Sumo protease was added in 1:100 mg (Ulp1: target protein) ratio and subjected to dialysis against 2 L of buffer C (50 mM Tris-HCl pH 8.0, 350 mM NaCl) overnight at 4°C. After overnight dialysis and cleavage of N-His tag, the protein was passed through a pre-equilibrated (buffer A) 5 ml HiTrap IMAC FF Nickel column (GE Healthcare). The flow-through was collected and the column was washed with buffer A and passed buffer B at a linear gradient of 150 ml of 100% buffer B. 4 ml fractions were collected and analyzed on a 15% SDS-PAGE. Flow-through and wash fractions from the above column were pooled and concentrated using a 30 kDa centrifugal concentrator (Amicon) and loaded onto a pre-equilibrated (with buffer D: 50 mM Tris-HCl pH 8.0, 150 mM NaCl) 120 ml Superdex200 16/600 column (GE Healthcare). 2 ml fractions were collected and analyzed on an SDS-PAGE gel. Fractions corresponding to pure PPAT protein were pooled and concentrated to 24 mg/ml, flash frozen in liquid nitrogen and stored at −80°C. The overall protein yield from a 6 L starting culture was approximately 100 mg. Identity of the purified protein was further confirmed by MALDI mass fingerprinting.

### Preliminary Fragment Screening

Thermal shift assays were carried out in a 96-well format with each well containing 25 μl of reaction mixture of 10 μM PPAT protein in buffer (50 mM Tris-HCl pH 8.0, 150 mM NaCl), 5 mM compound, 5% DMSO and 5x Sypro orange dye. Appropriate positive (Protein, DMSO and Coenzyme A) and negative (Protein, DMSO only) controls were also included. The measurements were performed in a Biorad-CFX connect thermal cycler using the following program: 25°C for 10 min followed by a linear increment of 0.5°C every 30 s to reach a final temperature of 95°C. The results were analyzed using Microsoft excel.

### Soaking of PPAT Native Crystals

Crystals for this experiment were grown at 19°C in 48-well sitting drop plates (Swiss CDI) in the following condition: 0.2 M Sodium bromide, 20%–24% PEG3350, 0.1 M Bis-Tris propane pH 6.5–7.5. Crystals were picked and allowed to soak in a 4 μl drop containing reservoir solution and 10 mM compound (in DMSO), which was then equilibrated against 250 μl of the corresponding reservoir solution overnight at 19°C in 24-well hanging drop vapour diffusion set up.

### Co-Crystallization of PPAT Protein

2 mM final concentration of compound in DMSO was added to 20 mg/ml of PPAT protein, mixed and incubated for 2 h on ice. Crystals were grown in the following condition: 0.2 M Sodium bromide, 20%–24% PEG3350, 0.1 M Bis-Tris propane pH 6.5–7.5 or in sparse matrix screens: Wizard 1&2 (Molecular Dimensions), Wizard 3&4 (Molecular Dimensions), JCSG + Suite (Molecular Dimensions). The crystallization drops were set up at a protein to reservoir drop ratio of 0.3 μl: 0.3 μl, in 96-well (MRC2) sitting drop plate, using Mosquito crystallization robot (TTP labtech) and the drops were equilibrated against 70 μl of reservoir at 19°C.

### Data Collection and Processing

X-ray data sets for PPAT apo and ligand-bound crystals were collected on I04, I03, I04-1 or I24 beamlines at the Diamond Light Source in the United Kingdom. The crystals were flash-cooled in cryo-protectant containing precipitant solution and 25% Ethylene glycol. The data sets were collected using the rotation method at wavelength of 0.979 Å, Omega start: 0^o^, Omega Oscillation: 0.15^o^, total oscillation: 240^o^, total images: 2,400, exposure time: 0.05 s. The experimental intensities were processed to 1.5 Å and the diffraction images were processed using AutoPROC (Vonrhein et al., 2011) utilizing XDS (Kabsch, 2010) for indexing, integration, followed by POINTLESS (Evans, 2011), AIMLESS (Evans and Murshudov, 2013) and TRUNCATE (French, 1978) programs from CCP4 Suite (Winn et al., 2011) for data reduction, scaling and calculation of structure factor amplitudes and intensity statistics. All crystals belonged to space group C2 2 21 and consist of three protomers in the asymmetric unit.

### Crystal Structure Solution and Refinement

The *Mycobacterium abscessus* PPAT ligand-bound structures were solved by molecular replacement using PHASER (Mccoy et al., 2007) with the atomic coordinates of *Mycobacterium abscessus* PPAT apo-structure (PDB entry: 5O06) as search model. Structure refinement was carried out using REFMAC (Murshudov et al., 2011) and PHENIX (Adams et al., 2010). The models obtained were manually re-built using COOT interactive graphics program (Emsley and Cowtan, 2004) and electron density maps were calculated with 2|Fo|- |Fc| and |Fo|–|Fc| co-efficients. Position of ligands in the protein active site and water molecules were located in difference electron density maps.

### Isothermal Titration Calorimetry (ITC)

ITC experiments were done using an ITC200 (MicroCal) instrument. The *Mab* PPAT protein for ITC was dialysed overnight at 4°C in storage buffer (50 mM Tris-HCl pH 8.0, 150 mM NaCl) the same buffer was used for preparing the ligand solutions. The protein and ligands/fragments were used at a concentration of 72 μM and 1–5 mM, respectively. ITC recorded for ATP was done in buffer: 50 mM Hepes pH 8.0, 150 mM NaCl, 5 mM MgCl_2_, at *Mab* PPAT concentration of 200 uM and 3 mM ATP. Buffer components and other constituents like DMSO were kept constant in both protein and ligand solutions. Ligands to buffer titrations were subtracted in all cases and experiments were repeated at least twice. Data were analysed using the Origin software (OriginLab, Northampton, MA, United States).

## Results

### *Mab* PPAT Binary Complex With Substrates ATP and 4′-Phosphopantetheine

To characterize the *Mab PPAT* ligand binding propensities, we determined crystal structures of *Mab* PPAT in complex with natural substrates ATP and 4′-phosphopantetheine (PhP). ATP occupies a positively charged pocket in the *Mab* PPAT substrate binding groove with the adenine ring making hydrogen-bond contacts with side chain hydroxyl of Thr118 (Figure 1A). The catalytic Arg90 at the base of the active site makes further H-bond and π-stacking interactions with the indole ring, while the adenine amino group is engaged in two additional hydrogen bonds with the backbone carbonyl groups of Val125 and Tyr122, respectively. The ATP ribose moiety mediates H-bonds to the side chain of Lys-87 and to three water molecules in the active site. The three phosphate groups of ATP are seen engaged in polar and H-bond interactions to the invariant catalytic residues Ser127, Ser128, Ser9, Phe10 and His-17, in addition to Mg^2+^ ion and several active site water molecules (Figure 1A).

In contrast to ATP, substrate 4′-phosphopantetheine (PhP) adopts a bent conformation in a predominantly hydrophobic part of the active site pocket. Here the 4′- phosphate group of PhP is engaged in a hydrogen-bond contact to the sidechain hydroxyl group of Ser9 and the terminal amide of the PhP β-mercaptoethylamine moiety makes further hydrogen bonds to the backbone nitrogen of Leu73 and side chain carboxylate of Glu132 at the edge of the active site. Major hydrophobic contacts in the region are mediated by Leu36, Pro7 and Leu73 at the top of the substrate site and Met101 and Leu129 at the base of the site. Figure 1C shows a detailed interaction map of the *Mab* PPAT- PhP complex.

### *Mab* PPAT Ternary Complex With PhP and AMPCPP: Insights Into Catalytic Cycle

Previous studies carried out on other bacterial PPAT orthologs have shown that the catalytic mechanism does not involve a covalent participation of residues in the functional site (Izard, 2002, Izard, 2003). Rather, the enzyme acts by providing the most favourable positioning of substrates to reduce the activation energy of the transition state. This allows the 4′-phosphate of 4′-phosphopantetheine (PhP) to undergo nucleophilic attack on the α-phosphate of ATP in an in-line displacement mechanism. The activation energy barrier of this reaction is decreased by PPAT by way of orienting the ATP β and γ-phosphates, thereby stabilizing the penta-covalent transition state (Izard, 2002; Izard, 2003). Here we determined, for the first time, a ternary complex of PPAT with 4′-phosphopantetheine and non-hydrolysable ATP analogue, AMPCPP. A superposition of the substrate-bound ternary structure of *Mab* PPAT with that of the reaction product dpCoA bound *Mab* PPAT structure, determined earlier (Thomas et al., 2017), shows close agreement in the positions of substrates and product (Figure 1D). 4′-phosphopantetheine (PhP) and the corresponding moiety in dpCoA overlaps perfectly in the PPAT active site. AMPCPP β and γ phosphates show greater flexibility in the corresponding crystal structure and therefore could be modelled in two different ways oriented away from the active site. The α-phosphate that undergoes nucleophilic attack by PhP could be identified unambiguously and is seen in close proximity (1.7 Å) to the α-phosphate of the reaction product dpCoA in the crystal structure, providing further evidence for the proposed catalytic mechanism (Figure 1D).

### *Mab* PPAT Complex With Feedback Inhibitor and Comparison With Product Binding

**FIGURE 2:**
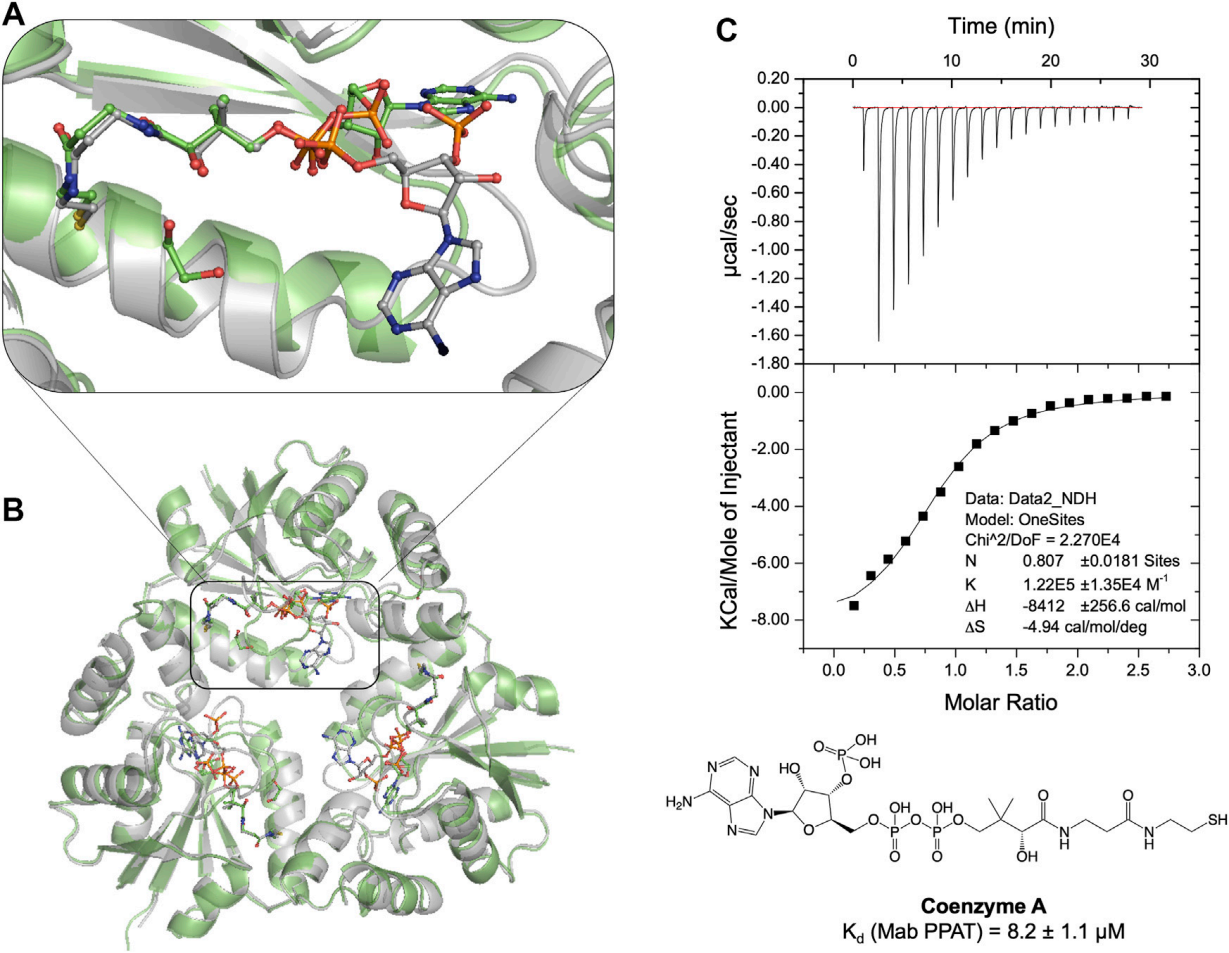
**(A)** Structural superposition of apo *Mab* PPAT in complex with feed-back inhibitor CoA (grey), PDB code 7YXZ and product dpCoA (green), PDB code 5O08, showing conformational differences in the enzyme and ligand binding mode. **(B)** The pantetheine moiety of CoA adopts a similar conformation to that of dpCoA. However, the CoA adenosine moiety is seen very flexible and largely solvent exposed in comparison to dpCoA in the *Mab* PPAT active site. **(C)** Thermodynamic (ITC) profile of CoA interaction with *Mab* PPAT.

Previous studies on *E. coli* PPAT have shown that Coenzyme A (CoA), the end-product of the biosynthetic pathway, acts as feedback inhibitor of the PPAT enzyme catalyzed reaction (Wubben and Mesecar, 2011). To further understand the binding mode of CoA, we determined the crystal structure of *Mab* PPAT in complex with CoA. CoA and the enzyme product 3′-dephospho CoA (dpCoA) differ only by the presence of a 3-phosphate on the ribose ring. A structural superposition of the *Mab* PPAT CoA structure with that of dpCoA bound form determined, previously within our research group (Thomas et al., 2017), shows that the pantetheine moiety of CoA and that of dpCoA adopts a similar conformation in the *Mab* PPAT active site (Figures 2A,B). On the contrary, the adenosine moieties of CoA and dpCoA do not coincide in the *Mab* PPAT active site. The adenine ring along with the 3′-phosphate group of CoA is found largely exposed to the central solvent filled channel in *Mab* PPAT-CoA structure whereas in *Mab* PPAT-dpCoA, the adenine ring adopts a different position near the catalytic Arg90 (Figures 2A,B). Further, the adenyl moiety of CoA exhibits high B-factors in comparison to that of the dpCoA complex, suggesting greater movements of this moiety in CoA-bound *Mab* PPAT. This could be attributed to the steric hindrance in the molecule imparted by the additional phosphate group of CoA. These observations further corroborate the earlier reports on PPAT- CoA structures from *E. coli* and *M. tuberculosis* (Morris and Izard, 2004; Timofeev et al., 2012), which show that the CoA adenine ring adopts a highly solvent-exposed conformation and/or exhibiting low electron-density.

### Fragment Screening Explores the Chemical Space of *Mab* PPAT Active Site

**FIGURE 3:**
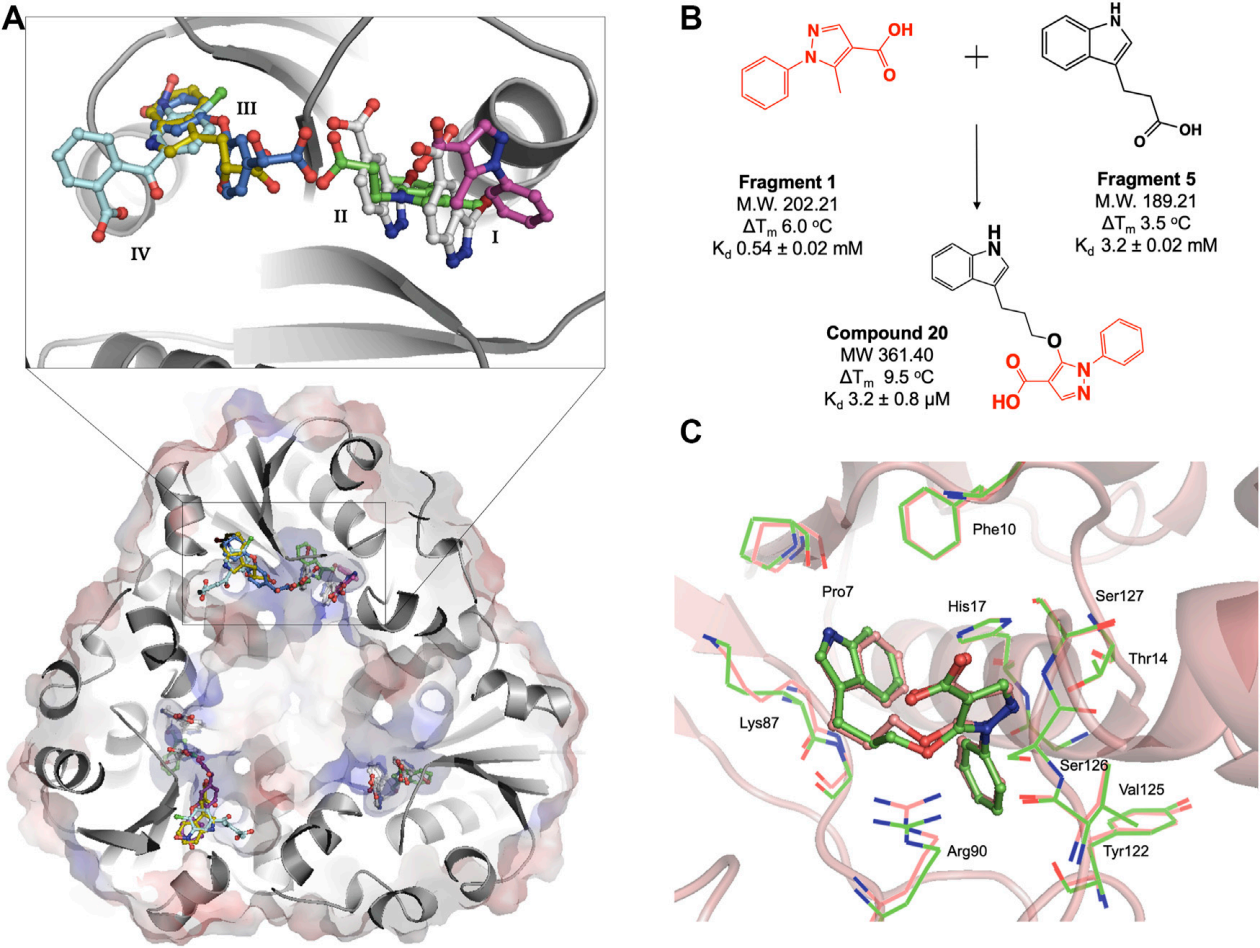
**(A)** Structural superposition of six representative *M. abscessus* PPAT crystal structures bound to fragments **1**(purple), **2**(white), **3**(green), **4**(blue), **5**(gold) and **6**(turquoise), showing fragments occupying four distinct regions in the PPAT active site. *Mab* PPAT is shown as surface electrostatic representation and grey cartoon model. **(B)** Fragment linking scheme leading to compound **20**, originally designed to target *M. tuberculosis* PPAT and the corresponding biophysical data (thermal shift (ΔT_m_) and binding affinity (K_d_) for *M. abscessus* PPAT ortholog, determined in this study **(C)** Superposition of compound **20** bound *Mab* PPAT (salmon) PDB code 7YYZ and *Mtb* PPAT (green), PDB code 6QMH structures showing close similarities in overall binding mode at the enzyme adenyl pocket.

Having identified the ligand binding modes and characterized the corresponding interactions, a fragment-based drug discovery campaign was explored to design inhibitors of *Mab* PPAT. The initial fragment screening of an in-house library (consisting of 960 fragments) was performed using fluorescence-based thermal shift assays. The hits obtained, exceeding a thermal shift cut-off of 3 standard deviations from the negative control (the PPAT and DMSO only), were further validated by X-ray crystallography and quantified using isothermal titration calorimetry (ITC). Preliminary results from this fragment screening effort have been described earlier (Thomas et al., 2017). A complete list of the validated fragment hits is shown in Table 1 and (Figure 3A and Supplementary Figure S1).

**TABLE 1:** Summary of fragment hits identified on *M. abscessus* PPAT shown along with corresponding thermal shift values and binding sites. Fragment chemical structures are also shown.

Fragment	PDB code	ΔΤ_m_ (°C)	Site	Chemical structure
1	5O0A	+6	**I**	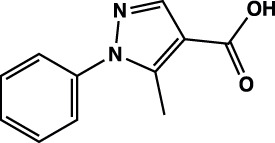
2	5O0B	+12.5	**I, II**	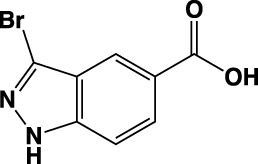
3	5O0C	+0.5	**II**	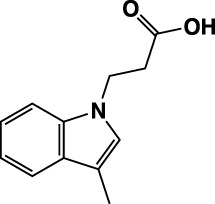
4	5O0D	+2.5	**III**	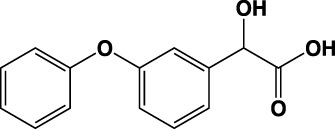
5	5O0F	+3.5	**III**	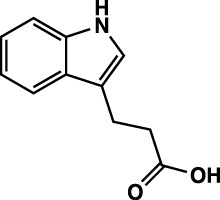
6	5O0H	+4.5	**III, IV**	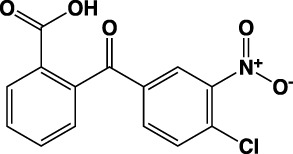
7	7YY3	+3.5	**III**	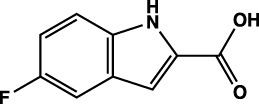
8	7YY4	+2.9	**III**	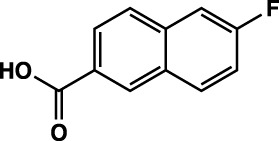
9	7YY5	+2.3	**III**	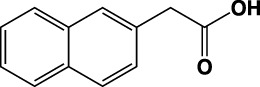
10	7YY6	+2.0	**I**	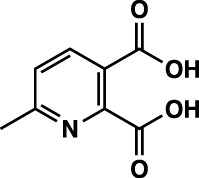
11	7YY7	+2.0	**III**	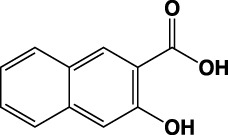
12	7YY8	+1.5	**III**	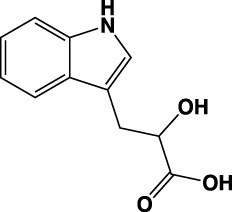
13	7YY9	+1.5	**I**	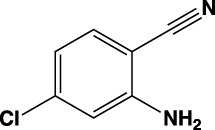
14	7YYA	+1	**II**	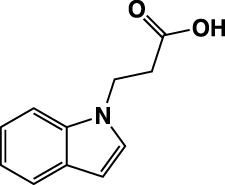
15	7YYB	+0.5	**III**	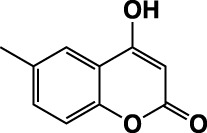
16	7YYC	-0.5	**II**	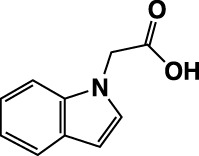

The fragment hits were shown by X-ray crystallography to occupy one of four sub-pockets in the CoaD active site, arbitrarily numbered **I—IV**, (Figure 3A). Sub-pockets **I** and **II** correspond to the ATP adenine and ribose phosphate binding regions of *Mab* PPAT. Fragments binding to this region engage in key interactions seen in the binding of *Mab* PPAT with ATP such as H-bond contacts with Thr118, Try122, Ser127, His17, Ser9 and π-stacking interactions with Arg90, His17 and Phe10, in addition to novel binding interactions mediated by Gly16 and Thr14 (Figure 3A and Supplementary Figure S1).

Fragment interactions at sub-pockets **III** and **IV** coincide with the binding mode of *Mab* PPAT in complex with PhP. Therefore, crucial contacts at this part of the active site, such as those mediated by Leu72-73, Leu36, Pro7, Ser9 and Glu132, were recapitulated by these fragments. Sub-pocket **III** is seen in an occluded state in apo, and native ligand bound *Mab* PPAT structures. Whereas fragment binding results in an open conformation allowing further chemical exploration of this cryptic site. Interestingly, many fragments are seen mediating additional π-stacking contacts such as those involving Phe76 and Tyr136 in this active site region (Figure 3A and Supplementary Figure S1). Identification of fragment hits were further informed by analysis using our in-house hotspot mapping program (Radoux et al., 2016). As described earlier (Thomas et al., 2017), this analysis performed on *Mab* PPAT apo structure, provided additional insights on how some of these fragment hits can be chemically elaborated into drug-like compounds.

### Evaluation of Fragment-Based Lead Compound Designed to Inhibit *M. tuberculosis* PPAT

While the chemical elaboration and lead optimization of the above fragments are currently underway (McCarthy, W.J., Thomas, S.E., *et al*, unpublished), we compared the fragment hits identified on *Mab* PPAT with the corresponding fragments studied earlier on PPAT ortholog from *M. tuberculosis* (sequence identity 77%). A multiple sequence alignment of PPAT homologues from seven bacterial species, including *M. tuberculosis*, reveals conservation of key amino acids with *Mab* PPAT. These include the proposed catalytic residues such as His17, Lys41, Arg90, Ser9 and Ser127 (Geerlof et al., 1999; Izard, 2002), as well as the highly conserved T/HxGH motif, the signature of the nucleotidyltransferase α/β phosphodiesterase superfamily (Supplementary Figure S2).

We therefore analysed the interactions of fragment hits, previously studied on *Mtb* PPAT, with respect to the corresponding *Mab* PPAT complexes. The binding modes of four different fragments were verified earlier on *Mtb* PPAT, using biophysical characterization and determination of crystal structures (El Bakali et al., 2020), of which the pyrazole fragment **3** recapitulates the binding mode adopted by the corresponding fragment (Fragment **1**) on the sub-pocket **I** of *Mab* PPAT (Figure 3A and Supplementary Figure S1). This fragment engages all the key contacts seen in the *Mtb* PPAT structure, such as H-bond to Thr14, Arg90, His17 and Ser127. Similarly, the Benzophenone fragment identified on *Mtb* PPAT (El Bakali et al., 2020) is involved in hydrophobic interactions with Leu73 and Leu36 and polar contacts to Asn105 and Glu132 as seen in the equivalent fragment analogue (Fragment **6**) on *Mab* PPAT sub-pockets **III** and **IV** (Figure 3A and Supplementary Figure S1). In contrast, unique binding modes were observed by fragments containing the indole moiety. For instance, in the case of *Mtb* PPAT, the indole fragments (**11** and **12)** bind solely at sub-pocket **II** of the active site chiefly mediated by active site His17 and Arg90 (El Bakali et al., 2020). While the corresponding fragments (**14** and **5**) in *Mab* PPAT occupy sub-pockets **II** and **III** respectively, engaging additional interactions through both hydrophobic interactions to Leu73 and Leu36 and polar contacts to Ser9 and Lys87 (Figure 3A and Supplementary Figure S1).

**FIGURE 4:**
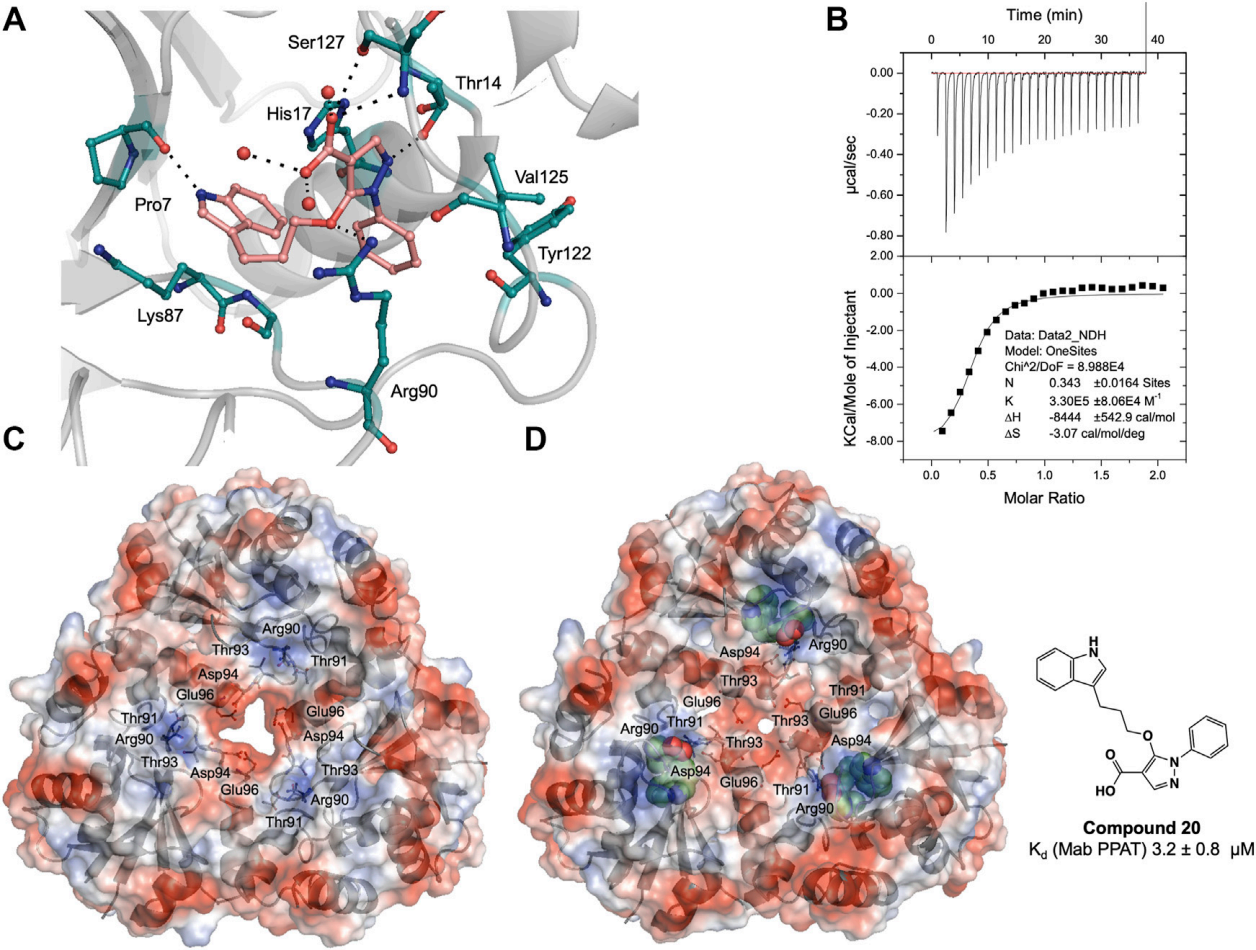
**(A)** Active site diagram showing binding interactions of compound **20** (salmon stick model) with *Mab* PPAT (grey). The interacting residues are shown as green stick representation. Polar hydrogen bond contacts are shown in black dotted lines and active site water molecules as red spheres. **(B)** Thermodynamic (ITC) profiles of compound **20** interaction with *Mab* PPAT. Surface electrostatic representation of *M. abscessus* PPAT illustrating **(C)** apo *Mab* PPAT (PDB code 5O06) having an open and predominantly negatively charged solvent channel and **(D)** Compound **20** (green spherical representation) bound form with closed conformation of the central solvent channel (PDB code 7YYZ). The key amino acid residues mediating the conformational switch are shown as grey stick model.

Having identified similarities and unique features in the fragment binding modes of *Mab* PPAT and *Mtb* PPAT, the question was asked whether the lead compound developed thereof against PPAT from *M. tuberculosis* would inhibit *Mab* PPAT, with comparable affinity. We therefore determined the crystal structure of lead compound **20** in complex with *Mab* PPAT and performed thermodynamic studies of the interaction (Figures 4A,B). Compound **20** was developed by linking of the pyrazole fragment **3** and the indole fragment **12** which bind at the *Mtb* PPAT sub-pockets **I** and **II,** respectively (El Bakali et al., 2020). As discussed earlier, although the corresponding prazole fragment **1** occupies sub-pocket **I** on *Mab* PPAT, the indole fragment **5** preferentially binds sub-pocket **III**, as do the indole fragments **7** and **12** on *Mab* PPAT. In contrast, three other indole fragments identified on *Mab* PPAT (**2, 3** and **16**), preferentially bind at sub-pocket **II** mediating interactions with His17, Arg90, Ser127, analogous to fragment **12** on *Mtb* PPAT (Figure 3A and Supplementary Figure S1).

Interestingly, the crystal structure of *Mab* PPAT in complex with compound **20**, solved in this study, shows similarities in binding mode and interactions, with that of *Mtb* PPAT at the ATP binding site (Figures 3C, 4A). The pyrazole part of the compound recapitulates interactions of the corresponding pyrazole fragment **1,** while the indole moiety, now positioned at sub-pocket **II**, makes H-bond contact with backbone carbonyl of Pro7 and cation-π interaction to Lys87. This change in binding preference of the indole moiety is not surprising, as interactions with this region of the active site are recapitulated by other indole fragments that occupy sub-pocket **II** as well as ATP. Furthermore, the overall affinity of the pyrazole moiety (fragment **1**, K_d_ 0.54 ± 0.02 mM), quantified by ITC on *Mab* PPAT, is higher than indole fragment represented by fragment **5** (K_d_ 3.2 ± 0.02 mM). The interactions gained by fragment **5** in sub-site **II** such as those mediated by the backbone carbonyl of Pro7 seems to compensate for those lost in sub-site **III,** such as hydrophobic contacts to Leu73 and Leu36, thereby further facilitating the alternate binding mode of the indole moiety of compound **20** (Figure 4A and Supplementary Figure S1).

### Compound 20 Results in Conformational Switching and Closure of Solvent Channel

As observed in PPAT from other bacterial species, *Mab* PPAT contains a solvent channel running through the centre of the hexamer along the 3-fold axis. Overlay of crystal structures of apo and compound-**20** bound to *Mab* PPAT shows that compound **20** results in large conformational change at the base of the PPAT active site and ordering of loop IV, connecting helix α4 and strand β4, to two complete α-helical turns. Such a dramatic shift is mediated by a change in orientation of Arg90 side chain which now makes hydrogen-bond and π-stacking contacts with the ligand and is no longer available to interact with Thr-91 and Thr-93 at the edge of the channel. The Arg-90 further makes polar contacts with the Asp-94 of the loop region thereby lowering the negative charge of the solvent channel. These conformational rearrangements of residues 89–96 exposed to the channel lead to closure of the PPAT solvent channel when bound to compound **20** (Figure 4D).

Similar rearrangements can be observed in ATP and dpCoA bound PPAT from Mab (Supplementary Figure S3) and other bacteria (Izard, 2003; Timofeev et al., 2012). Whereas in apo, PhP-bound and CoA-bound PPAT structures, the solvent channel adopts an open conformation and has a predominantly negative charge distribution from the acidic groups like Asp-94 and Glu-96 that are exposed to the channel (Figure 4C and Supplementary Figure S3). It was proposed earlier that these negatively charged residues lead to repulsion between protomers of the hexamer at the trimer interface playing an important role in subunit communication and in transitioning the enzyme from its symmetric to asymmetric forms. Additionally, the channel opening may also facilitate entry and binding of substrate to the active site (Morris and Izard, 2004; Timofeev et al., 2012). Therefore, the compound **20** bound *Mab* PPAT structure determined in this study presents an additional approach to enhance inhibition of the enzyme by blocking the central solvent channel, which may in turn prevent inter-subunit communication and access of substrates to the PPAT active site.

### Binding Affinity and Anti-Microbial Effect of Compound 20

Thermodynamic study of compound **20** binding to *Mab* PPAT shows a very significant improvement in binding affinity (K_d_ 3.2 ± 0.8 µM) by over two orders of magnitude in comparison to starting fragments (K_d_ ≥ 0.5 mM) (Figure 3B). An 80-fold improvement in the binding affinity is achieved by introduction of the linked indole ring. The affinity of compound **20** is comparable to that of *Mab* PPAT for feedback inhibitor CoA (K_d_ 8.2 ± 1.1 µM) (Figure 2C) and ≈ 7-fold greater than affinity of *Mab* PPAT for the substrate ATP (K_d_ 20.1 ± 3.3 µM) (Supplementary Figure S4). Notably, the binding affinity of compound **20** on *Mab* PPAT is nearly 5-fold higher than that on *Mtb* PPAT (K_d_ 15 ± 4 µM) (El Bakali et al., 2020).

However, compound **20** afforded only low cellular activity when profiled on *M. abscessus* ATCC19977 whole-cell cultures using a luciferase-reporter based luminescence assay up to a concentration of 200 µM in comparison to positive control, Amikacin (data not shown). NTMs, such as *M. abscessus*, are unique in their drug susceptibilities and ability to evade antibiotics through several powerful intrinsic resistance mechanisms. These include antibiotic-inactivating enzymes, target-modifying enzymes, drug efflux pumps and several additional genes acquired through horizontal gene transfer (Nessar et al., 2012; Nasiri et al., 2017). Further investigations are now underway to assess the lack of cellular activity of compound **20** and improve the physico-chemical properties of this early-stage lead compound.

## Discussion

In this study, phosphopantetheine adenylyltransferase (PPAT/CoaD) enzyme, involved in the coenzyme A biosynthesis pathway, was evaluated in the context of fragment-based discovery of new inhibitors targeting *Mycobacterium abscessus*. The structures of PPAT from *M. abscessus* in binary complex substrates 4′-phosphopantetheine (PhP) and ATP, feedback inhibitor Coenzyme A (CoA) and ternary complex with 4′-phosphopantetheine (PhP) and AMPCPP were solved at 1.5–1.8 Å resolution. These structures and corresponding thermodynamic studies provided detailed insights into the mode of ligand binding and catalytic process of *Mab* PPAT, facilitating the initiation of a structure-guided fragment-based drug discovery against this intrinsically drug-resistant *mycobacterium*.

Screening of a fragment library of 960 molecules by differential scanning fluorimetry and hit validation using X-ray crystallography resulted in the identification of 16 fragment hits. These fragment hits occupy four distinct sub-pockets in *Mab* PPAT, spanning the entire active site groove of the enzyme thus providing several potential starting points for drug discovery. While the chemical elaboration of the above fragments are on-going (McCarthy, W.J., Thomas, S.E., *et al*, unpublished), we investigated the compounds developed from a previous fragment-based drug discovery effort on *M. tuberculosis* PPAT ortholog (El Bakali et al., 2020), in the context of *M. abscessus*. The compound **20** studied was developed by linking of two fragments that occupy the PPAT adenylyl binding regions. This elaborated compound afforded low micromolar affinity against *Mab* PPAT with a dissociation constant of 3.2 ± 0.8 µM and the resulting crystal structures reveal important conformational changes imparted by the compounds on *Mab* PPAT. However, these compounds failed to show any significant inhibitory activity, when profiled on *M. abscessus* ATCC19977 whole-cell model. Further investigations are underway to assess whether the lack of cellular activity is related to permeability of the compounds across the thick cell envelope of mycobacteria or due to inactivation of compounds by the bacteria, to improve the physico-chemical properties of compound **20** and aid future drug discovery. The structural insights into substrate, feedback inhibitor and fragment binding modes described in this study can be utilized to further explore the chemical space of mycobacterial PPAT. In addition, the validated fragment hits identified in this study can also act as building blocks for new chemical entities targeting this essential bacterial enzyme. Overall, the study validates the ligandability of *M. abscessus* PPAT as an antibiotic target and identifies crucial starting points to facilitate structure-guided drug discovery against this highly-drug resistant *mycobacterium*.

## Data Availability

The datasets presented in this study can be found in online repositories. The names of the repository/repositories and accession number(s) can be found in the article/Supplementary Material. Coordinates and structure factors related to this work been deposited in the PDB with accession numbers 7YWM, 7YXZ, 7YY0, 7YY1, 7YY2, 7YY3, 7YY4, 7YY5, 7YY6, 7YY7, 7YY8, 7YY9, 7YYA, 7YYB and 7YYC.

## Abbreviations

PPAT, Phosphopantetheine adenylyl transferase; CoA, Coenzyme A; *Mab*, *Mycobacterium abscessus*; *Mtb*, *Mycobacterium tuberculosis*; ATP, Adenosine 5′ triphosphate; PhP, 4′-phosphopantetheine; dpCoA, 3′-dephospho CoA; NTM, nontuberculous mycobacteria; AMPCPP, Adenosine 5′-[α,β-methylene]triphosphate.
