# Supplementary material for: Structural Characterization of Mycobacterium abscessus Phosphopantetheine Adenylyl Transferase Ligand Interactions: Implications for Fragment-Based Drug Design
Source: Front Mol Biosci. 2022 May 30;9:880432. doi: 10.3389/fmolb.2022.880432 (PMC9197168; doi:10.3389/fmolb.2022.880432)
Supplement: Supplementary file 1 [file DataSheet1.docx]

**Supplementary Data**

**Structural characterization of *Mycobacterium abscessus* phosphopantetheine adenylyl transferase ligand interactions: Implications for fragment-based drug design**

Sherine E. Thomas1*, William J. McCarthy2, Jamal El Bakali2, Karen Brown3, So Yeon Kim1, Michal Blaszczyk1, Vítor Mendes1,3, Chris Abell2, R Andres Floto3,4, Anthony G. Coyne2 and Tom L. Blundell1*

1Department of Biochemistry, University of Cambridge, 80 Tennis Court Road, Cambridge
CB2 1GA, UK.

2 Yusuf Hamied Department of Chemistry, University of Cambridge, Lensfield Road, Cambridge, CB2 1EW, UK.

3Molecular Immunity Unit, University of Cambridge Department of Medicine, MRC Laboratory of Molecular Biology, Cambridge, UK.

4Cambridge Centre for Lung Infection, Royal Papworth Hospital, Cambridge, UK.

*Correspondence:

Sherine E. Thomas (set44@cam.ac.uk) or Tom L. Blundell (tlb20@cam.ac.uk)

**Present addresses:**

Sherine E. Thomas, Department of Pathology, University of Cambridge, CB2 1QP, UK.

William J. McCarthy, The Francis Crick Institute, London, NW1 1AT, UK

Jamal El Bakali, Univ. Lille, Inserm, CHU Lille, UMR-S 1172-LiNC-Lille Neuroscience & Cognition, F-59000 Lille (France)

Michal Blaszczyk, Cambridge Institute of Therapeutic Immunology and Infectious Disease, Department of Medicine, University of Cambridge, Cambridge, CB2 0AW, UK.


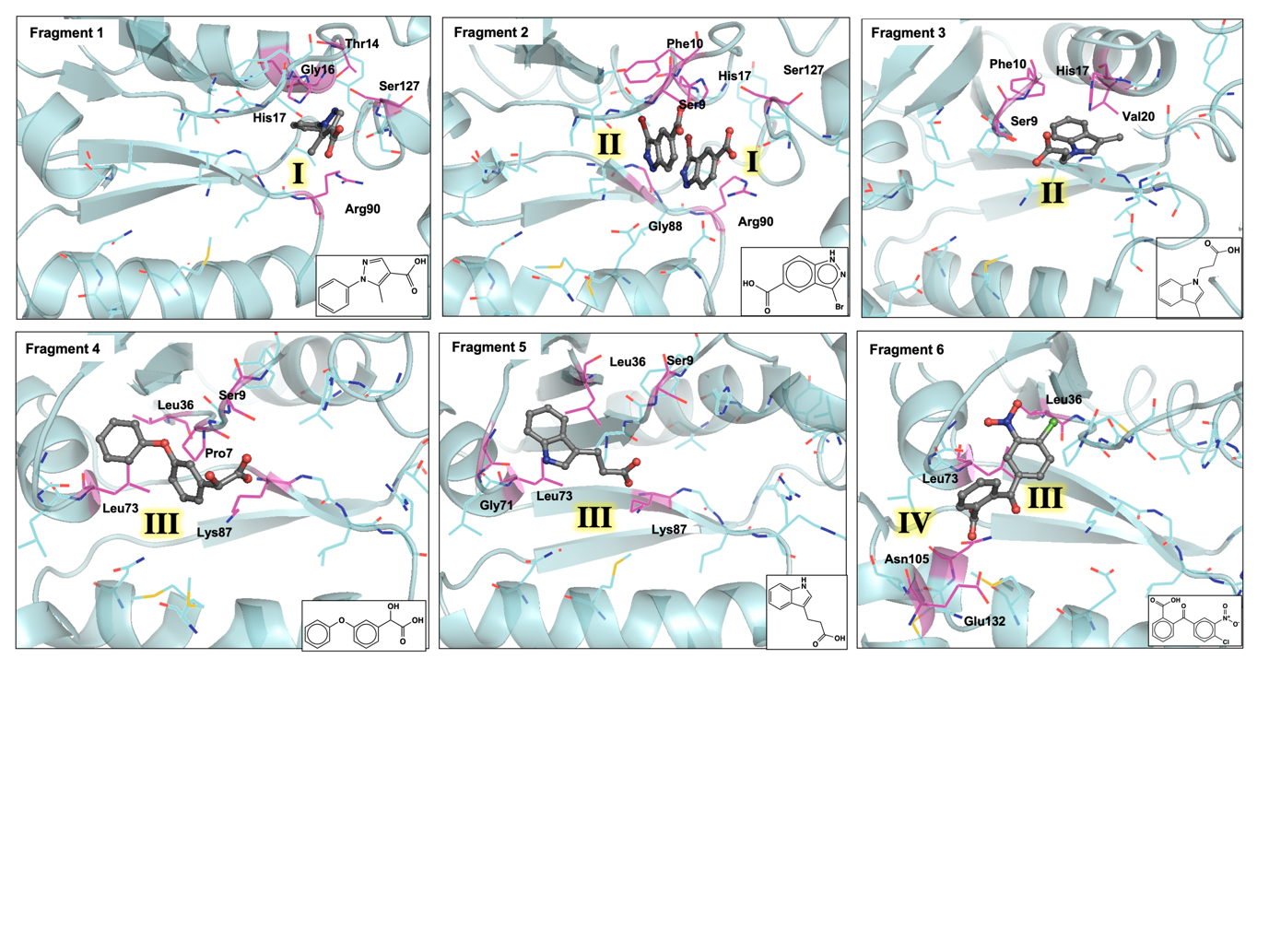


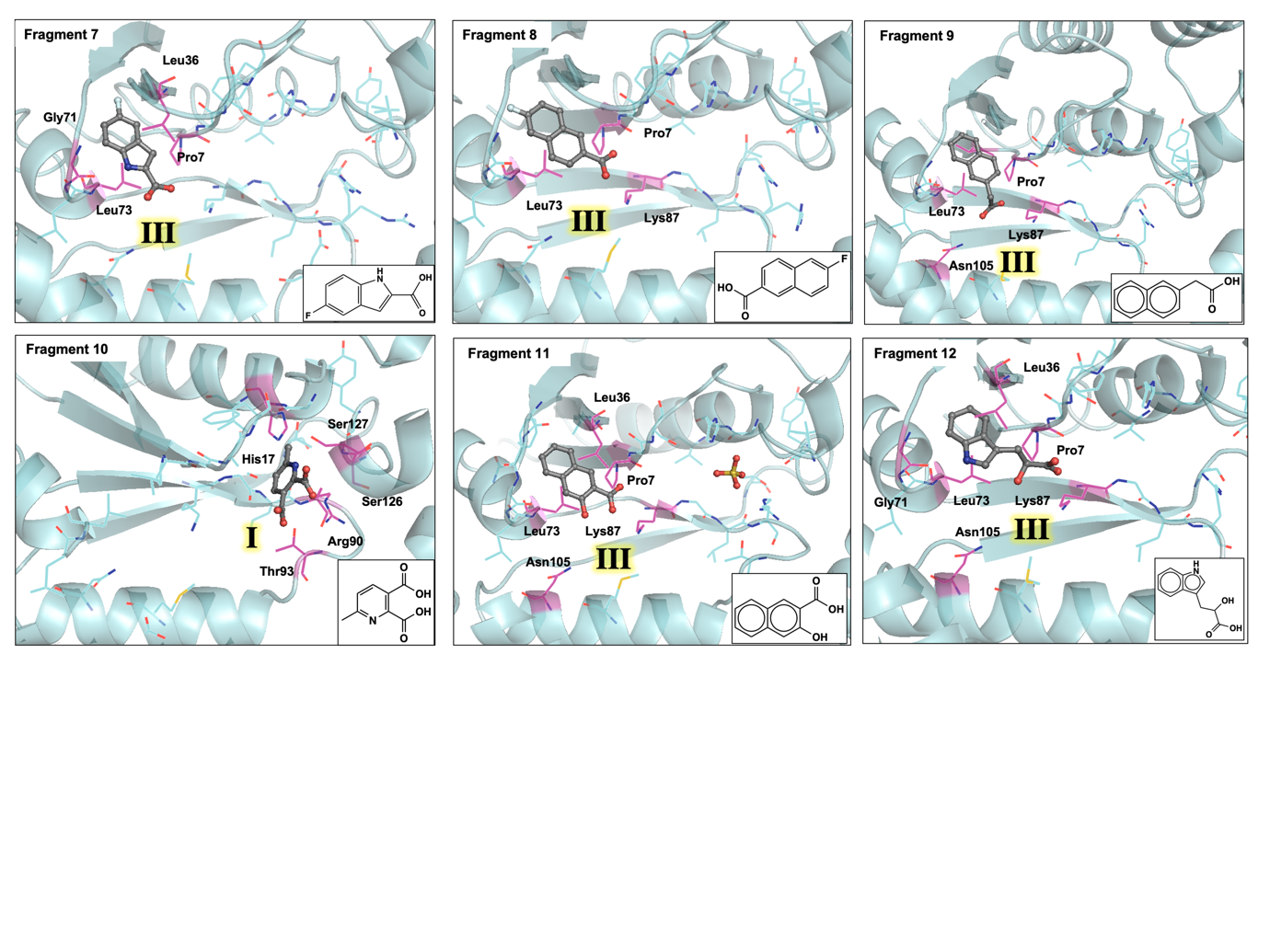


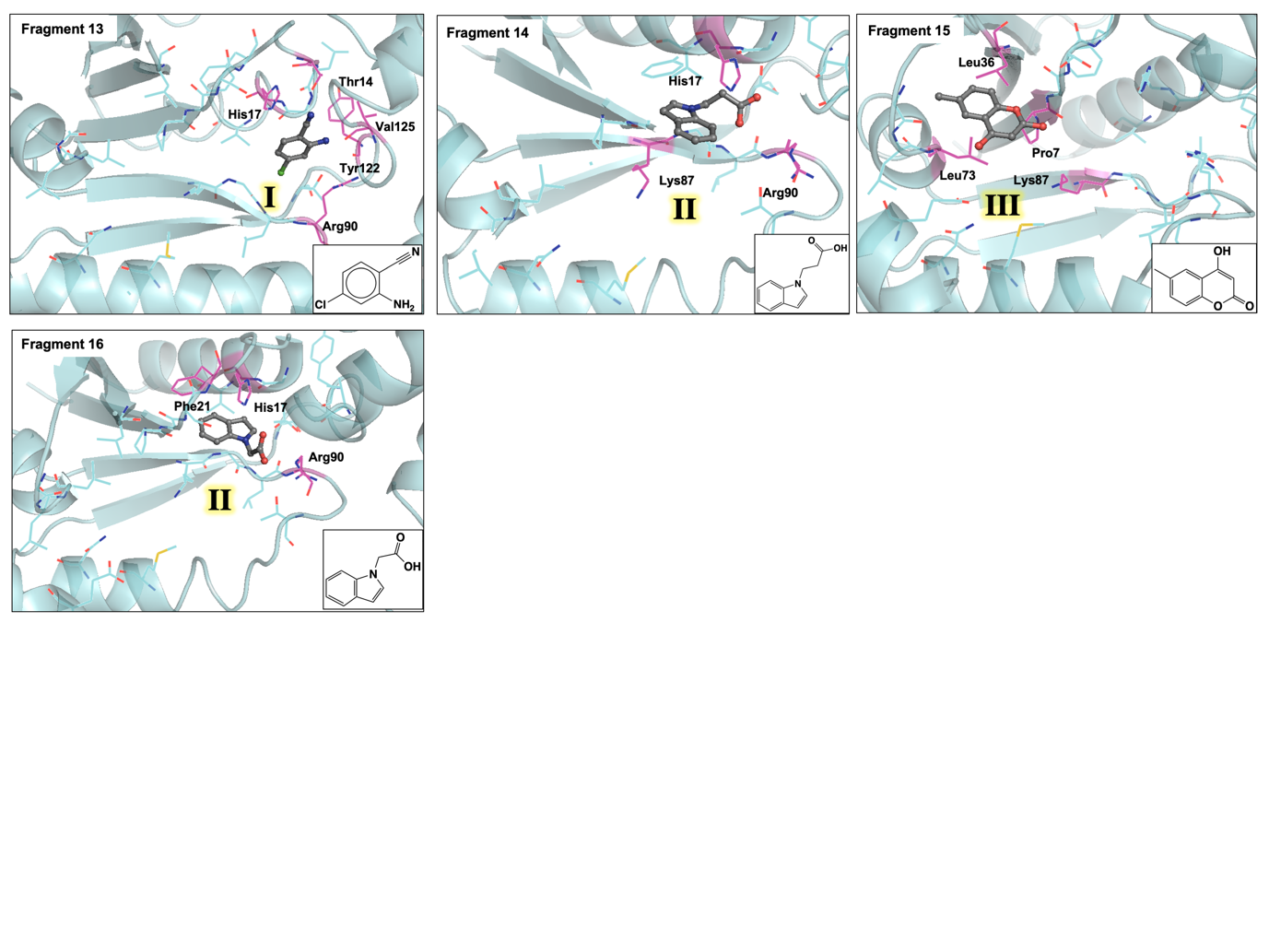


**Figure S1:** Crystal structures of *Mab* PPAT in complex with fragments showing key interactions mediated at sub-pockets **I-IV** of *Mab* PPAT active site. The PPAT protein is shown in blue, fragments as grey stick and interacting residues as pink line representation. The chemical structure of each fragment is also shown in the inset.


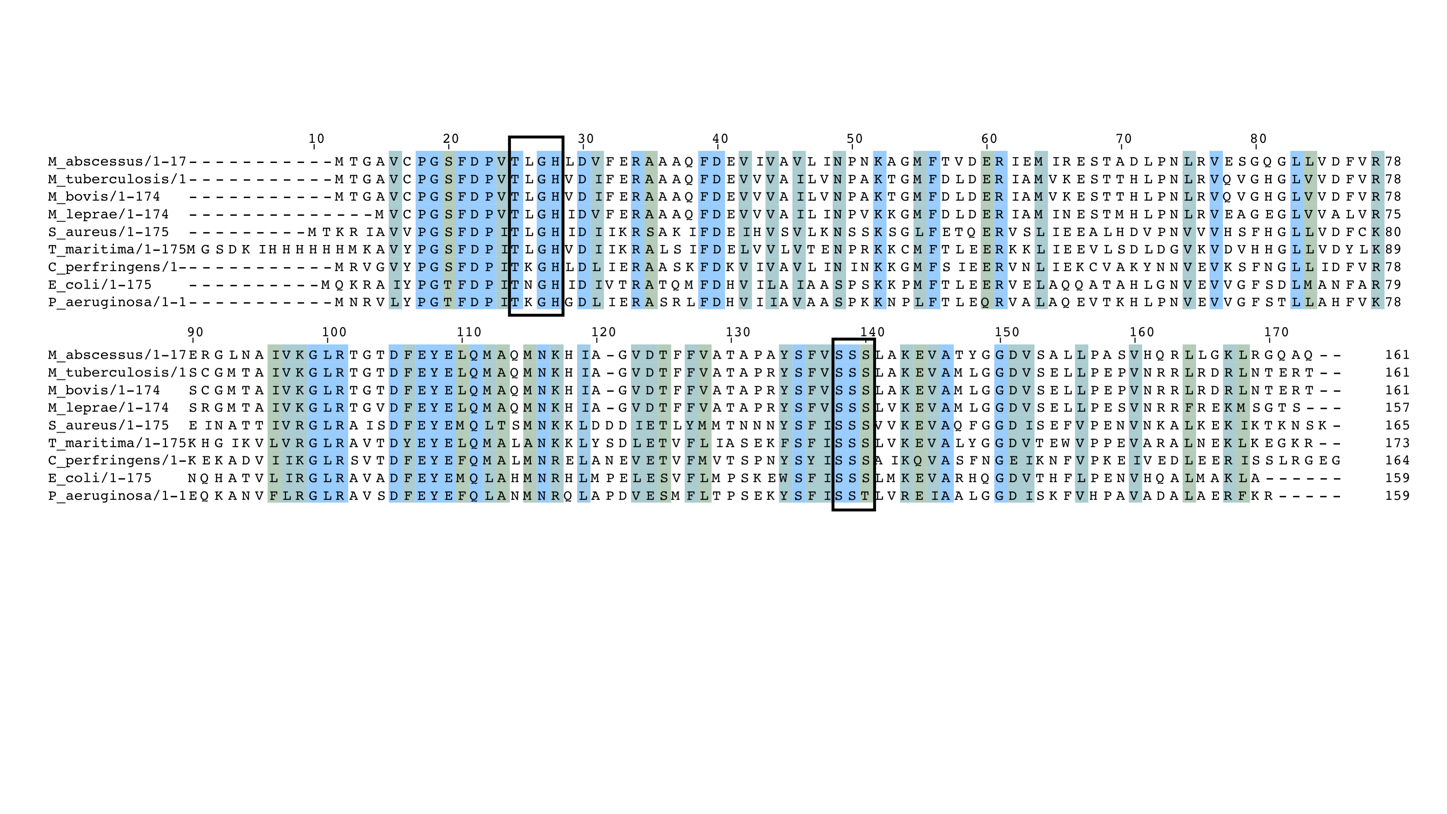


**Figure S2:** Multiple sequence alignment of nine PPAT homologs (*M. tuberculosis, M. bovis, M. leprae, T. maritima, S. aureus, C. perfringens, E. coli & P. aeruginosa*) with *M. abscessus* PPAT using CLUSTALW and analyzed using JalView showing conserved amino acid residues. The highly conserved T/HxGH motif and invariant Ser126-128 are boxed.


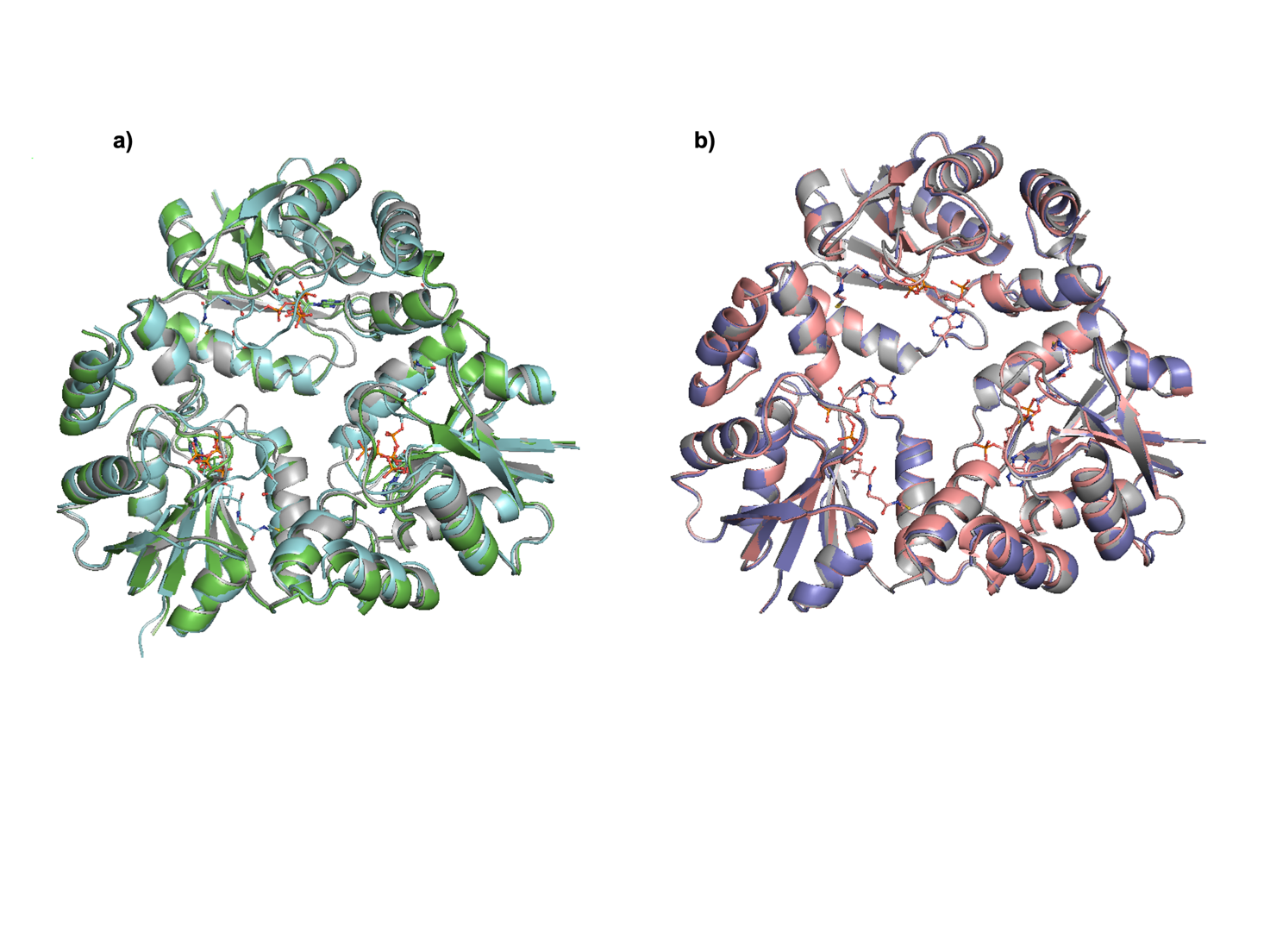


**Figure S3: a)** Overlay of crystal structure of *Mab* PPAT in apo (grey- PDB code 5O06) with ATP-bound (green-PDB code 7YWM) and product dpCoA- bound (blue -PDB code 5O08) forms showing conformational changes and ordering of loop IV at the base of the active site, in turn resulting in a closed sate of the solvent channel in ATP and dpCoA bound structures **b)** Superposition of crystal structure of *Mab* PPAT in apo (grey- PDB code 5O06) with substrate PhP-bound (purple- PDB code 7YY0) and feedback inhibitor CoA bound forms (salmon-PDB code 7YXZ), showing a conformation identical to that of *Mab* PPAT apo form where the solvent channel adopts an open state.


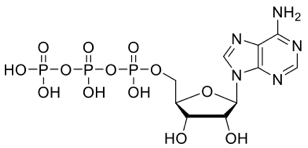

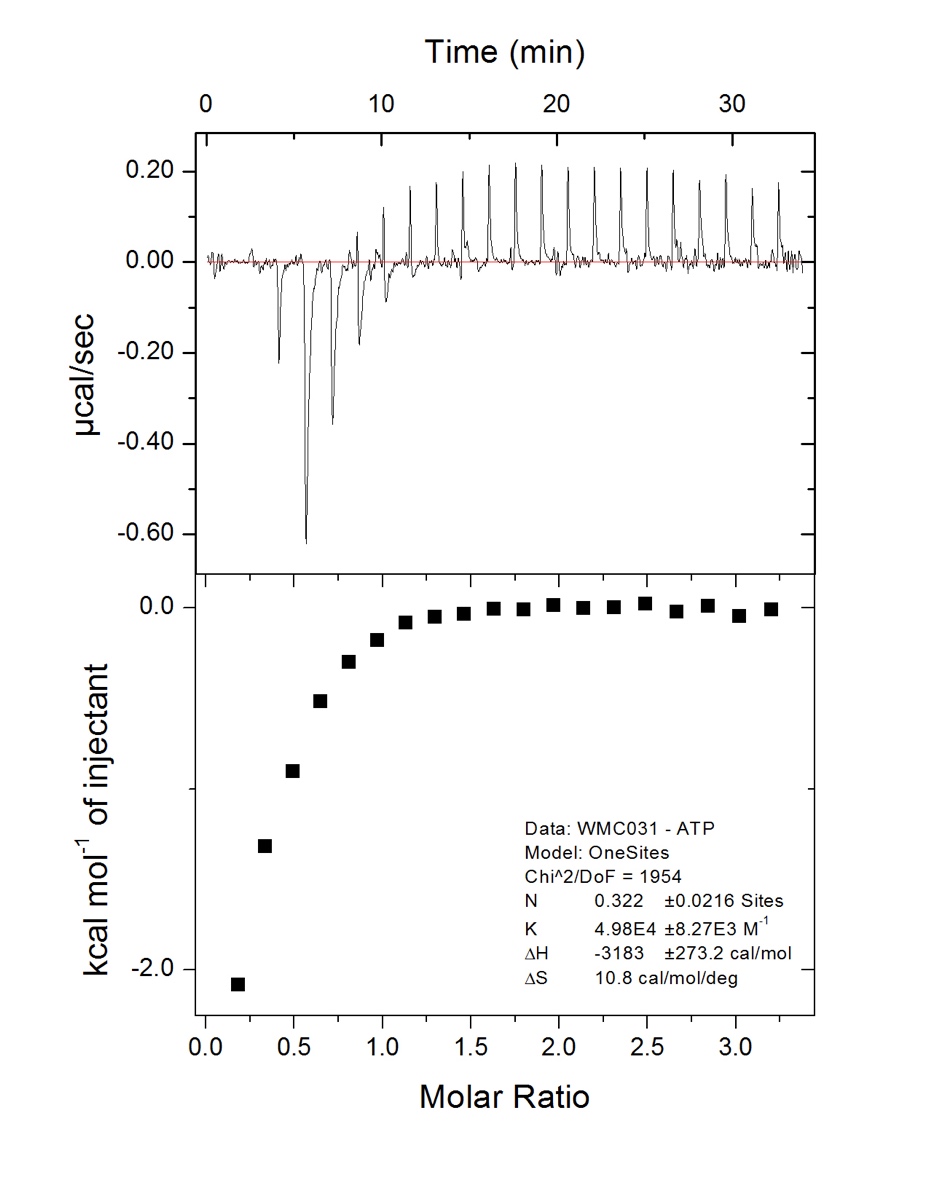


ATP

K_d_ = 20.1 ± 3.3 µM

**Figure S4:** Thermodynamic profile of the interaction of *Mab* PPAT with ATP

**Crystallography & Refinement Statistics**
